# Complete Genome Sequences of Four Natural *Pseudomonas* Isolates That Catabolize a Wide Range of Aromatic Compounds Relevant to Lignin Valorization

**DOI:** 10.1128/MRA.00975-20

**Published:** 2020-12-03

**Authors:** E. Anne Hatmaker, Gerald N. Presley, Olivia N. Cannon, Joshua K. Michener, Adam M. Guss, James G. Elkins

**Affiliations:** a Biosciences Division, Oak Ridge National Laboratory, Oak Ridge, Tennessee, USA; b Center for Bioenergy Innovation, Oak Ridge National Laboratory, Oak Ridge, Tennessee, USA; c Department of Microbiology, University of Tennessee, Knoxville, Tennessee, USA; University of Arizona

## Abstract

Many soil microorganisms have evolved catabolic strategies to utilize phenolic compounds arising from depolymerized lignin. We report the complete genome sequences of four *Pseudomonas* sp. isolates that demonstrated robust growth on a wide range of aromatic monomers and dimers that are relevant to the valorization of lignin into value-added chemicals.

## ANNOUNCEMENT

Lignin, a major structural component of plant cell walls, is one of the most abundant natural polymers. There is a growing interest in exploiting lignin as a renewable feedstock to produce a variety of value-added products, including bioplastics (1, 2), fungible fuels (3), and commodity chemicals (4), through chemical and biological valorization (5, 6). However, due to the complex nature of this heterogeneous aromatic polymer, a diverse range of enzymatic activities is required for depolymerization, aromatic ring opening, and conversion to target products (7–9). No bacterium has yet been isolated that can catabolize all components of depolymerized lignin. Instead, efficient valorization will likely require the isolation of additional bacteria with novel aromatic catabolism pathways, the rapid identification of the associated enzymes (10), and the heterologous expression of these enzymes in a production host (11).

To isolate new aromatic-catabolizing microbes, 50- to 100-ml broth enrichments were established by adding soils and river sediments as a source of inoculum at approximately 1.0% (wt/vol). These enrichments were grown in minimal M9 basal medium supplemented with 50 mg/liter cycloheximide and 0.1% (wt/vol) either ferulate (strains H1F5C and H1F10A) or dehydrodivanillic acid (strains B10D7D and B11D7D) at 30°C with shaking until turbidity was observed. Serial dilutions were plated onto M9 agar plates containing 0.1% (wt/vol) aromatic enrichment substrate as the sole carbon and energy source. Visible colonies were restreaked several times on the same medium for purification, and then a single colony was inoculated into 5 ml LB medium for DNA isolation, which was performed using a Quick-DNA fungal/bacterial microprep kit (Zymo Research, Irvine, CA). For strain identification, 16S rRNA genes were PCR amplified using primers 27F and 1492R (12), and the purified products were sequenced via the Sanger method (Eurofins Genomics, Louisville, KY). The sequences were aligned and compared to known sequences in the NCBI (nonredundant [nr]) and Greengenes databases using BLAST (13–15). Pseudomonas sediminis B10D7D and *Pseudomonas* sp. strain B11D7D were isolated from sediments (pH 7.0) collected from the Hiwassee River near Calhoun, TN (35.30000, −84.76397). Pseudomonas protegens H1F5C and H1F10A were isolated from acidic soil samples (pH 2.5) collected in the 100 Spring Plain area within Yellowstone National Park (YNP), WY (44.73323, −110.70976).

High-molecular-weight genomic DNA for genome sequencing was prepared from cells grown in LB broth using a protocol provided by the Joint Genome Institute (JGI) (https://jgi.doe.gov/wp-content/uploads/2014/02/JGI-Bacterial-DNA-isolation-CTAB-Protocol-2012.pdf). Pacific Biosciences (PacBio) SMRTbell library preparation (>10 kb, multiplexed) and long-read sequencing using the PacBio Sequel platform (Menlo Park, CA, USA) were performed by the Department of Energy Joint Genome Institute. Within the PacBio SMRT Analysis platform v5.0.1.9585, genomes were assembled from reads of >5 kb long using the Hierarchical Genome Assembly Process (HGAP) v4 (16) using the default settings. The modified sites that were detected were grouped into methylated motifs using MotifFinder (17) with default parameters. Genomes were annotated using the NCBI Prokaryotic Genome Annotation Pipeline (18), also with default parameters. All four *Pseudomonas* HGAP genome assemblies represent single, circular chromosomes with no plasmids (Table 1).

**TABLE 1:** Relevant genome sequencing and annotation statistics for *Pseudomonas* isolates

Species	Isolation location	Genome size (bp)	GC content (%)	Coverage (×)	No. of raw reads	No. of genes (PGAP)^*a*^	Raw read *N*_50_ (bp)^*b*^	SRA accession no.
*Pseudomonas sediminis* B10D7D	Calhoun, TN	4,934,017	62.4	194.0	674,756	4,612	63,091	SRX8889211
*Pseudomonas* sp. B11D7D	Calhoun, TN	5,387,171	62.5	180.0	219,370	4,985	62,275	SRX8889223
Pseudomonas protegens H1F5C	YNP, WY	6,818,519	63.1	330.0	498,582	6,214	51,295	SRX7717610
Pseudomonas protegens H1F10A	YNP, WY	6,817,972	63.1	213.0	219,370	6,213	56,328	SRX9016362

a PGAP, Prokaryotic Genome Annotation Pipeline.

b The *N*_50_ value was calculated based on genome size for each isolate.

Methylated motifs were found in two of the genomes. Two methylated motifs were found in *Pseudomonas* sp. B11D7D—TGGANNNNNNNRTNGC, consistent with type I restriction-modification (RM) systems (19), and CATGRAG. The *P. sediminis* B10D7D methylome also includes one type I RM, CAANNNNNTCGC, and a second motif, CCGCGAG. Underlined bases represent adenine methylation on the forward or reverse strand.

**FIG 1:**
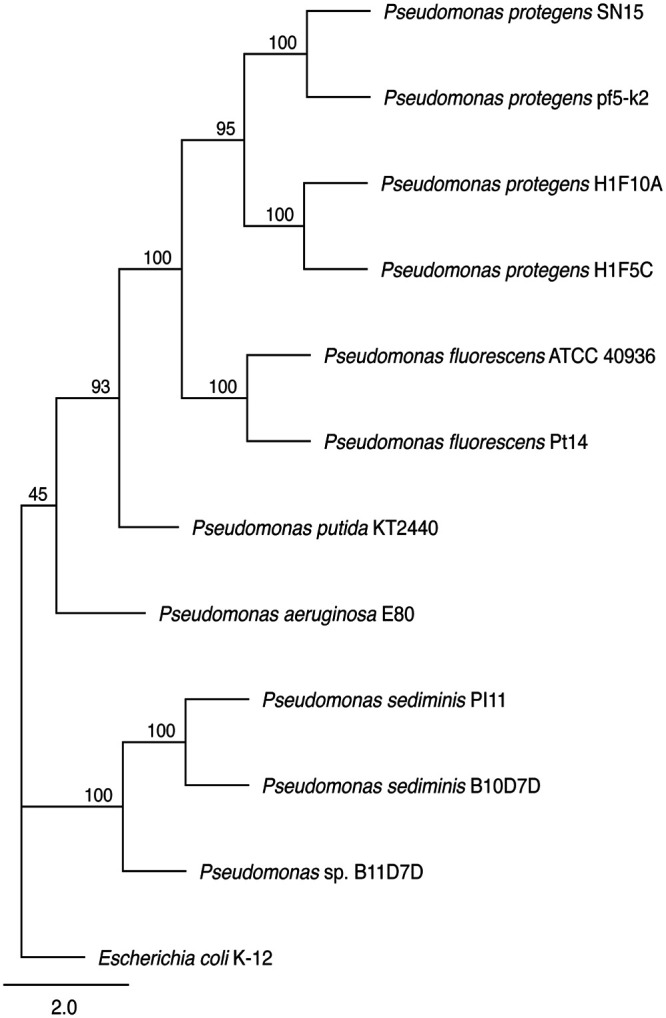
Maximum likelihood phylogeny of *Pseudomonas* isolates from this study and other relevant *Pseudomonas* strains and E. coli K-12. Numbers above nodes represent bootstrap values, which were calculated from 1,000 replicates with 10% burn-in.

Using the single-copy gyrase B (*gyrB*) gene, we constructed a maximum likelihood phylogeny of the *Pseudomonas* isolates and RAxML and the GTR+GAMMA model with 1,000 replicates for bootstrapping (20), showing the relationship between our isolates and commonly studied species (Fig. 1). Complete genome assemblies enable systems biology studies and genetic engineering, facilitating future studies of these lignin-degrading isolates.

### Data availability.

The complete genomes for strains B10D7D, B11D7D, H1F5C, and H1F10A were deposited in GenBank under accession numbers CP060009, CP060008, CP060201, and CP060289, respectively.
